# The potential for senotherapy as a novel approach to extend life quality in veterinary medicine

**DOI:** 10.3389/fvets.2024.1369153

**Published:** 2024-05-15

**Authors:** Zoë J. Williams, Lyndah Chow, Steven Dow, Lynn M. Pezzanite

**Affiliations:** ^1^Department of Clinical Sciences, College of Veterinary Medicine and Biomedical Sciences, Colorado State University, Fort Collins, CO, United States; ^2^Department of Microbiology, Immunology and Pathology, College of Veterinary Medicine and Biomedical Sciences, Colorado State University, Fort Collins, CO, United States

**Keywords:** senotherapeutics, senescence-associated secretory phenotype, dasatinib, quercetin, fisetin

## Abstract

Cellular senescence, a condition where cells undergo arrest and can assume an inflammatory phenotype, has been associated with initiation and perpetuation of inflammation driving multiple disease processes in rodent models and humans. Senescent cells secrete inflammatory cytokines, proteins, and matrix metalloproteinases, termed the senescence associated secretory phenotype (SASP), which accelerates the aging processes. In preclinical models, drug interventions termed “senotherapeutics” selectively clear senescent cells and represent a promising strategy to prevent or treat multiple age-related conditions in humans and veterinary species. In this review, we summarize the current available literature describing *in vitro* evidence for senotheraputic activity, preclinical models of disease, ongoing human clinical trials, and potential clinical applications in veterinary medicine. These promising data to date provide further justification for future studies identifying the most active senotherapeutic combinations, dosages, and routes of administration for use in veterinary medicine.

## Introduction

**Figure 1:**
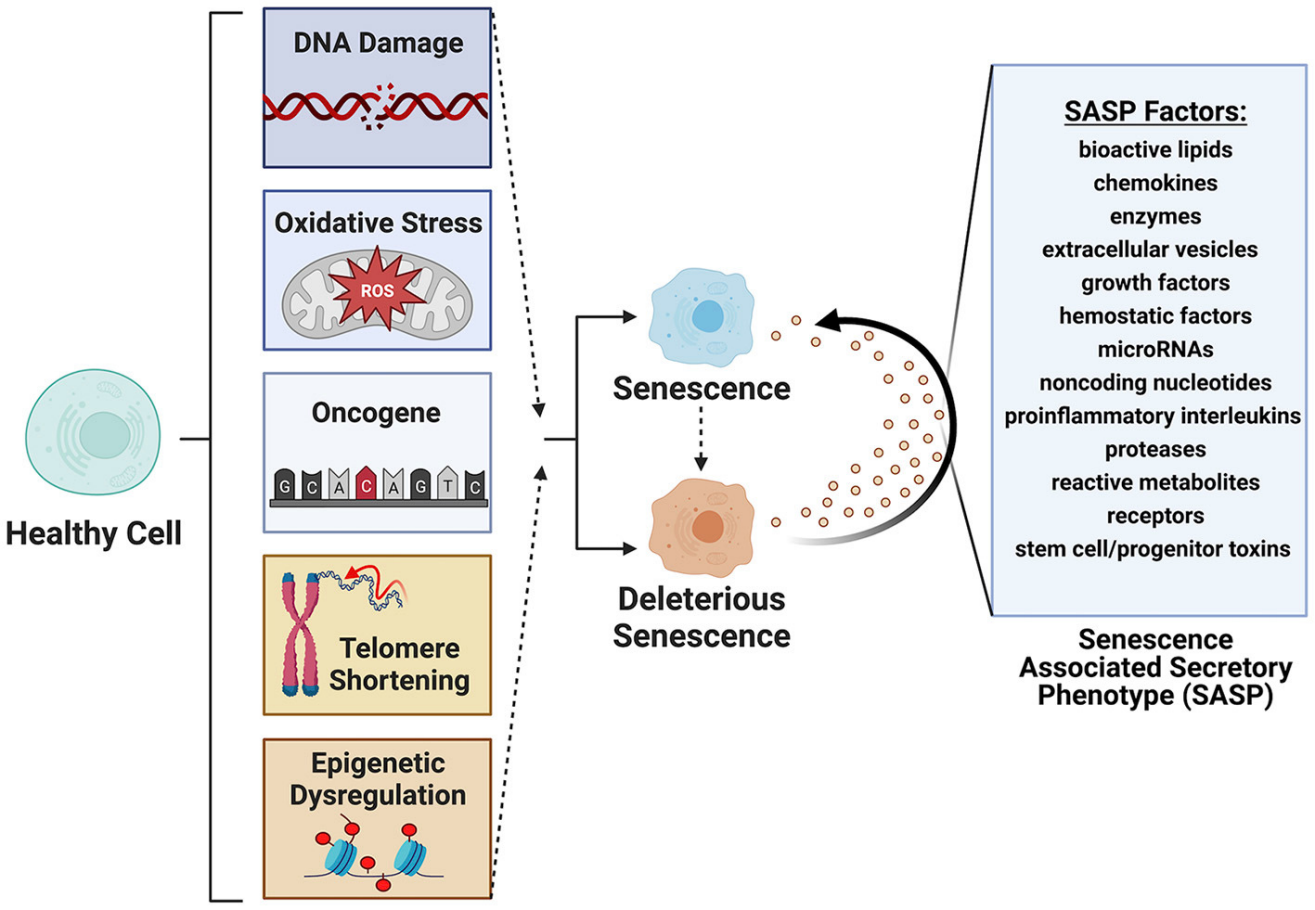
Senescence is induced by factors such as DNA damage, oxidative stress, oncogene formation, telomere shortening and epigenetic modifications. Deleterious senescent cells produce a senescence associated secretory phenotype (SASP) with paracrine or juxtacrine signaling of factors that promote deleterious senescent states in adjacent cells. Created with BioRender.com.

Cellular senescence refers to the physiological mechanism by which proliferating cells undergo stable cell cycle arrest upon stress or damage and secrete an array of factors, termed the senescence associated secretory phenotype (SASP) (1–5) (Figure 1). Cellular senescence is a hallmark of aging (6, 7), first documented in 1961 by Hayflick and Moorehead (8). In health, senescent cells (SC) are cleared from tissues by innate immune and natural killer cells (9, 10). In disease, these cells can accumulate beyond the capacity of immune surveillance mechanisms to fully clear. This massive expansion of senescent cells is driven by paracrine and endocrine signaling mechanisms that induces a senescence state in neighboring cells. A portion of senescent cells (between 30 and 70%) are termed deleterious senescent cells and can secrete inflammatory, pro-apoptotic, insulin resistance-inducing cytokines (e.g., TNF-α, IL-6,) and chemokines (MCP-1, IL-8) that attract and activate innate immune cells. Other effects include activation of matrix metalloproteinases that cause tissue destruction, bioactive lipids that contribute to inflammation (e.g., prostaglandins, bradykinins), microRNAs that contribute to stem and progenitor cell dysfunction, and extracellular vesicles (EVs) carrying cytotoxic cargo to neighboring cells (3, 11–13).

Cellular senescence underlies numerous age-related diseases (e.g., congestive heart failure, strokes, Alzheimer's, cancer, metabolic dysregulation, renal dysfunction, chronic lung diseases, osteoporosis, osteoarthritis) (3, 14) as the SASP phenotype initiates immune clearance driving chronic inflammation (15, 16). Consequently, a popular rationale for senotherapy is to reduce the overall senescent cell burden. This in turn would lead to reduced inflammation, decreased macromolecular dysregulation and improved function of stem and progenitor cells (2, 17, 18). These approaches, including the various senotherapeutics under development, their mechanisms of action, and early preclinical or clinical evidence of activity are the subject of this review.

## Detection and induction of cellular senescence *in vitro*

While general hallmarks of cellular senescence have been established, including structural, epigenetic and signaling alterations, a universal set of sensitive and specific markers has yet to be agreed upon, which complicates senescence studies (3, 4, 19). Understanding the mechanisms leading to senescence allows for the detection and experimental induction of cellular senescence. SC exhibit a complex gene expression profile, marked by increased levels of p16^Ink4a^ and p21^Cip1^, along with the activation of pathways that help them resist apoptosis, known as senescent cell antiapoptotic pathways (1, 20). Detection of cellular senescence may be performed through detection of primary markers of cell cycle arrest. These markers are characterized by upregulated expression of protein markers p16 (p16^INK4a)^, p21 (p21^Cip1^), p53, decreased phosphorylated Retinoblastoma protein (pRB), and structural changes associated with increased lysosomal content and senescence-associated ß-galactosidase (SA-ß-Gal) (21, 22). Two separate, yet interconnected, pathways that drive cell cycle arrest are p53/p21 and p16/pRb (16, 23, 24). The p53 regulated gene product p21, plays a role in arresting the cell cycle through inhibition of cyclin-dependent kinase-2 (CDK2) inactivation retinoblastoma (Rb) protein necessary for cell cycle progression (23). Through the inhibition of cyclin-dependent kinase 4 (CDK4) and CDK6, p16^INK4a^ prevents the phosphorylation of the Rb protein (23, 24). The expression of markers such as p53, p16^INK4a^, and p21^Cip1^ increase during senescence, making them commonly used indicators for senescence (25, 26).

Secondary senescence biomarkers include flattened or enlarged cellular morphology and SASP expression (e.g., IL-6, IL-8, IL-1a, GRO-a, MCP-1, and IFN-γ, among others) (4). Detecting SASP markers does not verify senescent cell states outright, as many factors other than senescence can trigger SASP cytokine secretion such as infections, immune-mediated diseases, and cancer. Profiling senescent cells with gene and protein expression analyses has demonstrated upregulation of antiapoptotic pathways in certain types of neoplastic processes such as lymphocytic leukemia and B-cell lymphoma (27, 28). Efforts toward defining the pleiotropic nature of senescent cells and SASP signatures are ongoing and have recently been published for specific cell lines (29), which warrants further investigation utilizing cell lines isolated from veterinary species of interest.

**Figure 2:**
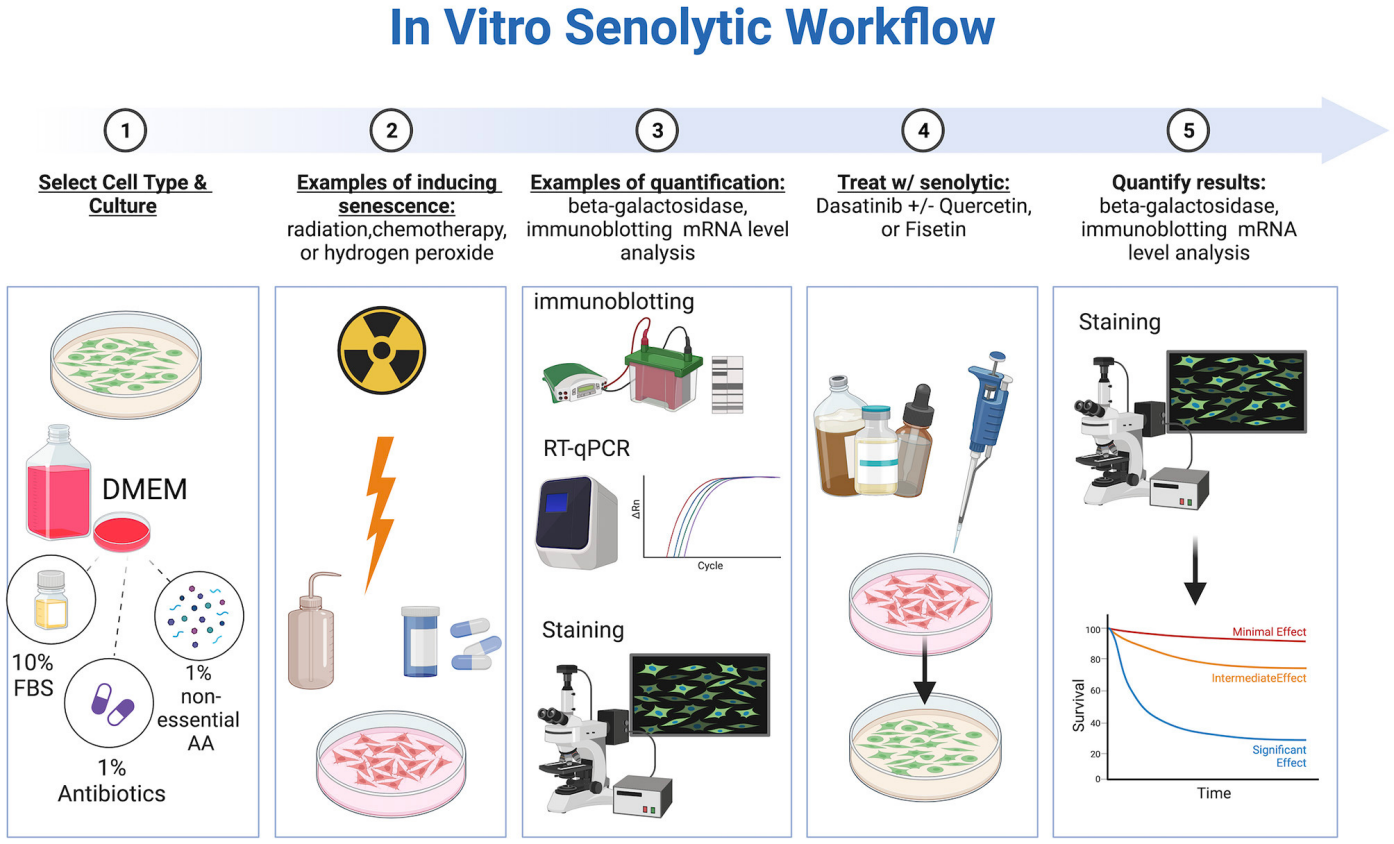
Workflow of *in vitro* senotherapeutic drug studies comprised of cell culture, induction of cell senescence, senotherapeutic treatment, and final quantification with statistical analyses. Created with BioRender.com.

*In vitro* studies evaluating senotherapeutics have integrated various cell lines including WI-38, IMR-90, mouse embryonic fibroblasts (MEFs), human preadipocytes, human umbilical vein endothelial cells (HUEVCs), and bone marrow-derived mesenchymal stem cells (BMD-MSCs) with artificially induced senescence (30–33). Fibroblasts, in particular, are commonly used in studying overall aging mechanisms (34). Multiple methods to induce senescence have been reported including a single dose of gamma-radiation (e.g., 10 Gray), exposure to bleomycin, chemotherapy drugs, hydrogen peroxide, TGF-β1, oncogene induction, or replicative exhaustion (8, 33, 35, 36). The optimal method to induce senescence depends on the cell line evaluated (37). Specific considerations regarding *in vitro* evaluation of senotherapeutics include concentration, duration of exposure, and solubility. To identify and quantify senescent cells (SCs), several techniques have been described, including SA-ß-Gal staining, gamma-histone 2AX (γ-H2AX), senescence associated heterochromatin foci markers, immunoblotting for senescence-associated proteins, mRNA level analysis, CDKN2A/p16, and detection of senescence-associated secretory phenotype proteins (38, 39) (Figure 2). Of note, cells can adopt a senescent phenotype simply under regular culture conditions, which is indicated by elevated levels of SA-β-Gal, the DNA damage marker γ-H2AX in the nucleus, and a rise in the expression of p21 mRNA (40).

Preluding gene expression, epigenetic regulators modulate transcription senescent markers and SASP factors (41, 42). This was shown in human stromal cells when histone H3-specific demethylase KDM4 was found to potentiate senescent markers such as p16^INK4a^, p21^CIP1^, and CXCL8 (43). Another study evaluated the epigenetic modifications of preadipocytes in first degree relatives of people with type 2 diabetes (44). When compared with controls, those individuals had increased senescent marker expression and hypomethylation of a gene called *ZMAT3*. Increased protein expression due to hypomethylation, led to premature senescence. This suggests an epigenetic influence on development of senescence and potential predisposition to type 2 diabetes (44). In summary, while general indicators of cellular senescence have been established, a complete set of sensitive and specific markers has yet to be agreed upon, which complicates detection of senescence and monitoring response to treatment with senotherapeutics, representing an area for further investigation.

## Preclinical evidence for senotherapeutics

Multiple different therapeutic approaches to remove senescent cells have been investigated following the initial reports that caloric restriction or mutations that decreased growth hormone signaling significantly extended lifespan, presumably in part through reduction of the senescent cell burden (3, 45). Since then, over 40 compounds have been identified that target senescent cell associated anti-apoptotic pathways (SCAPs) with the potential for use as senotherapeutics (30). From these screens, priority was given to drugs that targeted multiple pathways, could be administered orally, and were natural products with either known safety profiles or already approved by the US Food and Drug Administration (FDA) for use in humans (1). Three such drugs, including the tyrosine kinase inhibitor dasatinib and the natural flavonoids quercetin and fisetin, were identified as promising (1). These senotherapeutics have consequently been investigated in multiple recent and ongoing human clinical trials for treatment of diseases such as osteoarthritis, skeletal health, mobility, and frailty, among others (1). Here we present a summary of the currently described senotherapeutic drugs for potential application in veterinary medicine.

### First generation senotherapeutic drugs

First-generation senotherapeutics and their activities are summarized in Table 1 and described in further detail here.

**Table 1:** Summary of available first and second generation senotherapeutic drugs.

**1st generation**					
**Senotherapeutic**	**Mode of action**	**Efficacy**	***In vitro*** **dose**	**Notes**	**References**
Dasatinib (D)	1. SRC/tyrosine kinase inhibitor 2. Interferes with EFNB-dependent suppression of apoptosis	1.↓ viability + cell death of senescent human preadipocytes 2. Less effective on human umbilical vein cells (HUVECs) compared to preadipocytes	50 nM: ↓preadipocyte viability by 30–40% 250 nM: ↓ Ercc1-deficient murine embryonic fibroblasts (MEFs) 500 nM: not efficacious in ↓ bone marrow-derived mesenchymal senescent cells (BM-MSCs) from progeroid Ercc1–/Δ mice		(30–32)
Quercetin (Q)	1. Inhibits PI3K 2. Inhibition of mTOR Signaling 3. Represses plasminogen activator inhibitor-1 4. Inhibits serpines	1. ↓ viability + caused cell death of senescent HUVECs 2. Less effective on preadipocytes compared to HUVECs	10 μM: ↓HUVEC SC by 50% 50 μM: ↓Ercc1-deficient MEFs 100 μM: ↓sc of BM-MSCs from progeroid Ercc1–/Δ mice		(30, 46, 47)
D + Q	^*^See above^*^	Selective killing of both senescent preadipocytes (↓65%) and endothelial cells	D: 200 nM Q: 20 μm		(30)
Fisetin (F)	1. BCL-2/BCL-xL inhibitor 2. HIF-1α inhibitor	1. Effective at reducing senescent markers in MEFs, human adipocytes 2. Induces apoptosis in HUVECs 3. NOT senolytic in IMR90 cells or primary human preadipocytes	0.5 μM: caspase activity ↑ 5 μM: ↓cell viability (Best supported) 10 μM: ↓ cell numbers	Low toxicity	(48, 49)
Navitoclax (ABT263)	BCL-2/BCL-xL inhibitor	Depletes senescent bone marrow hematopoietic stem cells & senescent muscle stem cells	0.313 μM: ↓cell viability of SC 5μM: ↓cell viability of *non*-SC 1.25 μM: *in vitro* and *vivo* ↓~50% viability after 5 h exposure	Hematological toxicity (platelets and immune cells)	(50)
Luteolin	Modulation of SIRT1 and p53	1. Weak activity on MEFs SCs (5 μM) 2. Rescues 50% of H_2_O_2_ induced SC in House Ear Institute-Organ of Corti 1 (2 μM)	2 μM−5 μM		(49, 51, 52)
Curcumin	Targets NF-kB, MAP-kinase, p53, NRF2, AKT, COX-2 and EGFR	1. Weak activity on murine embryonic fibroblast SCs (5 μM) 2. No significant senolytic activity at sublethal doses (<10 μM)	5 μM−10 μM	↓Bioavailability, water-insoluble, significant cytotoxic + genotoxic effects at ≥ 10 μM	(49, 53, 54)
Curcumin Analog EF24	1. Induces apoptosis 2. Reactive oxygen species (ROS) production 3. Proteasome degradation of the Bcl-2 family proteins	1. EC50of 1.62 μM in SC induced by radiation 2. EC50 of 4.69 μM in non-SC	EF24 had minimal effect on the cell viability of WI-38 NCs <4 μM		(55)
A1331852	BCL-2 family member inhibitors	1. Induces apoptosis in senescent HUVECs & IMR90 cells 2. Not senolytic against human preadipocytes	1 nM	Appears less toxic than navitoclax	(48)
A1155463	BCL-2 family member inhibitors	1. Induces apoptosis in senescent HUVECs & IMR90 cells 2. Not senolytic against human preadipocytes	1 nM	Appears less toxic than navitoclax	(48)
Geldanamycin/Alvespimycin (17-DMAG)	HSP90 inhibitors	↓ Viability of SC ME (1 μM) without significantly affecting non-SC	1 μM (EC50 for SC 7 nM; EC50 for non-SC 73 nM)	Alvespimycin is more water soluble	(53)
Tanespimycin (17-AAG)	HSP90 inhibitors	↓ Viability of SC ME (1 μM) without significantly affecting non-SC	1 μM		(53)
Piperlongumine	Induces apoptosis, mechanism unknown	Kills senescent human WI-38 fibroblasts & IL1β induced senescent goat chondrocytes	10 μM for 48 h (EC50 for SC 6.24–7.97 μM; EC50 for non-SC 20.28 μM) 5 μM ➔mild chondrocyte death after 3 days 10 μM ➔cytotoxicity 3–7 days	N-acetylcysteine inhibits piperlongumine	(56, 57)
FOXO4-related peptide	Targeted apoptosis of senescent cells by p53 nuclear exclusion	Kills senescent primary human IMR90 fibroblasts	25 μM for 3 days		(58)
Nutlin3a	MDM2 inhibitor	Kills senescent melanoma cells and retinal pigment epithelial cells	2.5–10 μM	Dose dependent cytotoxicity Can INDUCE senescence	(59, 60)
Cardiac Glycosides	Na+/K+ATPase pump inhibitors	Kills senescent human fibroblasts IMR90, osteoarthritic chondrocytes,	~0.1 μM		(61, 62)
Aspirin	COX2 inhibitor	Kills doxorubicin induced senescent human fibroblasts, murine embryonic fibroblasts, and amyloid induced human neuronal cells	100 μM		(63)

### Dasatinib

Dasatinib is an FDA approved tyrosine kinase inhibitor used to treat chronic myeloid leukemia and acute lymphoblastic leukemia, as well as refractory non-Hodgkin lymphoma, metastatic gastrointestinal stromal tumors, and metastatic prostate cancer in humans (64, 65). Dasatinib functions as a senotherapeutic by interfering with ephrin ligand dependent suppression of apoptosis (30–32). When administered, dasatinib decreases viability of senescent human preadipocytes by 30–40% (30) at concentrations of 50 nM and murine embryonic fibroblasts (MEFs) at concentrations of 250 nM (30). However, with an apparent cell line specific effect, dasatinib has not demonstrated efficacy against senescent human umbilical vein cells (HUVECs) nor senescent bone marrow-derived mesenchymal cells (30–32), which prompted evaluation of co-administration with other senotherapeutics, such as quercetin, to enhance efficacy.

### Quercetin

Quercetin is a naturally occurring flavonoid found in certain fruits, vegetables, onions, and kale. Quercetin inhibits kinases and serpines integral to modulating cell growth and arrest such as phosphoinositide 3-kinases (PI3K) (46, 47). Clinically, quercetin alone has cardioprotective effects (66, 67) by reducing obesity, restoring plasma thyroid hormone levels, mitigating cardiac oxidative stress (66, 68), and promoting angiogenesis (69). ApoE-/- hypercholesterolemic mice that were treated with quercetin had a significant reduction in left ventricular (LV) hypertrophy (70). Quercetin enhanced antioxidant defenses and improved cardiac bioenergetics in rats that were fed a high fat diet (67). Likewise, quercetin attenuated oxidant-induced endothelial dysfunction in high cholesterol mice fed a high fat diet by enhancing nitric oxide bioavailability (71). Mouse atherosclerosis studies have shown variable pro-inflammatory and non-specific systemic effects of both genetic and pharmacological senolysis (72). While quercetin has demonstrated efficacy to induce senolysis on senescent HUVECs, MEFs, and BM-MSC *in vitro*, but with limited significant therapeutic effect on preadipocytes (30, 46, 47), demonstrating an efficacy profile opposite that of dasatinib. Therefore, quercetin has been investigated further in combination with dasatinib (3, 14, 30, 73–80).

### Dasatinib and quercetin

The combination of dasatinib and quercetin has proven effective in mice at a dosage of 5 mg/kg of dasatinib and 50 mg/kg for quercetin (30, 81), and clears from systemic circulation within 48 h after the last dose (82, 83).

Relevant to ***cardiovascular disease***, dasatinib and quercetin has demonstrated significant improvements in vascular smooth muscle sensitivity to nitroprusside and an enhanced ventricular left ejection fraction in mouse models of cardiac disease (84). This senotherapeutic combination also activates cardiac progenitor cells following treatment in a mouse model of cardiovascular disease (85). Both aged mice and hypercholesterolemic mice treated with dasatinib and quercetin had significant reductions in telomere associated foci, staining of aortic calcification, and osteogenic gene and protein expression (86). Another study demonstrated that dasatinib and quercetin treatment not only reduced SC numbers, but also promoted cardiomyocyte regeneration in aged mice (85). In an arteriovenous fistula chronic kidney disease mouse model, dasatinib and quercetin treatment significantly reduced p16^Ink4a^ venous expression, indicating reduction in SC (81). Pre-treatment with this senotherapeutic combination has also been shown to reduce the quantity of SC in arterial walls and lessened the severity of abdominal aortic aneurysms in mice given angiotensin II (87).

Relevant to ***obesity***, evaluation in mouse models have indicated that the combination of dasatinib and quercetin reduces inflammation, alleviates metabolic dysfunction pre-adiposity, and facilitates the differentiation of adipose cells into mature, insulin-responsive ones (88). Aged mice treated with dasatinib and quercetin had decreased SA-ß-Gal, p16 and p21 expression in white adipose tissue. Dasatinib and quercetin also suppressed age-related increases in pro-inflammatory SASP genes (*MCP-1, TNA-*α*, IL-1*α*, IL-1*β*, IL-6, CXCL-2*, and *CXCL-10)* (76). That study also demonstrated improved fasting blood glucose, glucose tolerance, reduced plasma triglycerides, and improved systemic lipid tolerance in old mice treated with dasatinib and quercetin (76). Human adipocytes treated with dasatinib and quercetin demonstrated reduced insulin resistance following xenotransplanation. Because insulin resistance is a risk factor of type 2 diabetes, these findings indicate that senotherapeutics may offer a new treatment avenue to target adipocytes expressing elevated p21^Cip^ (89).

With respect to ***neurologic disease***, mouse models of Alzheimer's disease have found that Tau-containing neurofibrillary tangles display senescent phenotypes with increased CDKN2A expression and brain atrophy (90). When 23-month-old tau transgenic mice were treated with dasatinib and quercetin, neurofibrillary tangle burden, ventricular enlargement, and neurodegeneration was significantly reduced (90). Furthermore, treatment with dasatinib and quercetin in a mouse model of Alzheimer's showed reduction of SC in amyloid-β plaques, reduced neuroinflammation (IL1-β, IFN-γ and TNFα), and significantly improved cognitive function (91). Another study investigating dasatinib and quercetin to treat age-related cognitive decline in male Wistar rats found that this therapeutic combination alleviated learning deficits, memory impairment, and markers of peripheral inflammation (IL-1α, IL-β, IL-4, IL-2, IL-10, MCP-1 and TNF-α) (92). Furthermore, these differences were associated with cellular changes to dendritic spine morphology, specifically hippocampal CA1 neurons and molecular alterations of histone H3 trimethylation at lysine 9 and 27 (92). While quercetin alone was found to ameliorate the progression of intervertebral disc disease in mice via the Nrf2/NF-κB axis (93), the combination of dasatinib and quercetin have shown decreases in p16^INK4a^, p19^ARF^, IL-6, and MMP13 expression while preserving cell viability (94). The neuroprotective effects of dasatinib and quercetin have also been demonstrated in a controlled cortical impact mouse model of traumatic brain injury over the time of 4 months (95). Treatment with dasatinib and quercetin significantly reduced SASP pro-inflammatory factors, IL-1β, IL-6, and attenuated neurodegeneration in mice with cortical impact traumatic brain injury (95). Furthermore, clinical behavior testing of treated mice revealed significantly improved spatial reference memory and improvement in depression-like behavior (95). Following treatment with dasatinib and quercetin, obese mice showed improved neurogenesis and a significant decrease in anxiety-like behavior (96). Specifically, dasatinib and quercetin treatment increased Nestin-positive neuronal precursor cells, double-courtin positive immature neurons, and CD133 positive ependymal cells (96).

Relevant to ***pulmonary disease***, in a mouse model of idiopathic pulmonary fibrosis, dasatinib and quercetin treatment significantly improved lung compliance (97). This was further supported by a study evaluating dasatinib, quercetin, or dasatinib and quercetin in a mouse model of hyperoxia induced airway smooth muscle senescence (98). Mice treated with quercetin (25 mg/kg) had improved pulmonary compliance but not resistance, while dasatinib (1 mg/kg dasatinib) improved both compliance and resistance (98). To model premature neonatal pulmonary disease, fetal smooth muscle explants exposed to moderate hyperoxia (40% O_2_) for 7 days display significant increases in senescent markers such as SA-ß-Gal, p16, p21, p53, and the DNA damage marker gamma-H2AX (99). Following treatment with dasatinib and quercetin, there was a reduction in cells expressing SA-ß-Gal, p21, p16, and phosphorylated γ-histone family member X (99).

With respect to ***renal disease***, treatment with dasatinib and/or quercetin reduced renal tubular senescence and ameliorated renal fibrosis in multiple mouse models of acute kidney injury (100). In models involving a high-fat diet and renal fibrosis, treatment quercetin alone decreased SC burden, reduced protein secretome markers (p16, p19, and p53), and improved renal function shown by a decreased plasma creatinine (101).

Relevant to ***orthopedic disease***, one study found that treatment with dasatinib and quercetin decreased osteoclastic activity while promoting osteoblastic differentiation in mouse models of osteoporosis (102). Conversely, in another mouse model (accelerated aging Z24–/– model) dasatinib and quercetin treatment did not mitigate trabecular bone loss (103) while another study indicated dasatinib and quercetin treated mice may actually improve bone production of aged bone marrow derived mesenchymal stem cells (104). Dasatinib and quercetin treatment have facilitated growth of aged muscles through SC clearance and altered *Igf1, Ddit4, Mmp14* gene expression in a mouse model of blunted muscle hypertrophy (105).

Finally, dasatinib and quercetin have been investigated in several other unrelated disease processes in rodent models. In a murine model of sclerodermatous graft vs. host disease, the administration of a combination of dasatinib and quercetin led to a reduction in peripheral senescence-associated secretory phenotype (SASP) cytokines, specifically IL-4, IL-6, and IL-8Rα (106). In a mouse model of doxorubicin-induced ovarian injury, both dasatinib and quercetin and fisetin treatments reduced SC presence, however treatment was unable to restore normal ovarian function (107). A study conducted in aged mice revealed that dasatinib and quercetin treatment significantly decreased SC markers p16 and p21 expression, as well as the expression of inflammatory markers Cxcl1, Il1β, Il6, Mcp1, and Tnfα in the small and large intestine (108). Notably, dasatinib and quercetin treatment also induced alterations in specific microbial signatures across ileal, cecal, and colonic regions, and within feces.

### Fisetin

Fisetin is a natural flavonoid with several reported modes of action that target the anti-apoptotic pathways of senescent cells, including BCL-2/BCL-xL inhibition (48, 109) and induction of hypoxia–inducible factor−1α (HIF-1α) (3, 110). Promoting apoptosis through increased caspase activity (48, 49), fisetin successfully targeted senescent HUVECs (48). Studies have shown that treatment with 500 nM fisetin induces apoptosis in senescent cells derived from subcutaneous adipose tissue (48) and that the effect is larger in adipose cells expressing *p16*^*Ink*4*a*^ and lower in *p21*^*CIP*1^-expressing cells (49).

*In vivo*, fisetin has been studied at dosages ranging from 60 to 100 mg/kg, administered orally for up to five consecutive days, exhibiting efficacy in reducing the number of senescent cells in white adipose tissue. One study evaluated the impact of fisetin on neuronal cellular senescence in a randomized controlled trial using aged sheep (111). There was a significant decrease in senescent neurons, astrocytes, and microglia in the neural tissue of sheep treated with fisetin (111). The treatment group also exhibited decreased senescent mRNA expression of SA-ß-Gal in the lung, heart, and spleen. Furthermore, the senescent marker p21 was also decreased in the liver and lung, and inflammatory markers in the lung, liver, heart and spleen following treatment (111).

A mouse model of systemic lupus erythematosus found that senescent neural cells accumulate in a hippocampal region of the brains of mice with depressive behavior (112). Oral administration of fisetin successfully reduced the number of senescent neural cells, reduced depressive behavior and limited SASP factors in the hippocampal region (112). Mouse models of chronic wounds also support a potential benefit to the senotherapeutic fisetin in chronic wound management and elimination of dermal SC (113). Targeting SC, treatment with fisetin improved muscle function and myogenic phenotypes in a mouse model of muscular dystrophy (114). Conversely, in a mouse model of chronic inflammatory myopathy, a benefit to senescent fibro-adipogenic-progenitor cells was demonstrated in mitigating exercise-induced muscle degeneration through pro-inflammatory regenerative mediators while mice without senescent adipogenic-progenitor cells exhibited muscle degeneration, which warrants further investigation (115).

### Navitoclax

Navitoclax (ABT263) is a BCL-2 inhibitor with demonstrated efficacy to reduce the number of senescent bone marrow hematopoietic stem cells and muscle stem cells (50). Navitoclax reduced the senescent cell burden in HUVECs, IMR90 human lung fibroblasts, and MEFs by targeting BCL-2 proteins (116). However, navitoclax has reported side effects of neutrophil toxicity and thrombocytopenia (50, 117, 118). To mitigate these adverse effects, galacto-conjugation of navitoclax has shown a significant reduction in platelet toxicity and increased senotherapeutic specificity in both human blood samples and a murine lung cancer model (119). Explant studies have also shown that human pulmonary endothelial cells from patients with pulmonary arterial hypertension have a significant increase in SC burden and these cells undergo apoptosis when exposed to navitoclax *in vitro* (120).

Relevant to ***cardiovascular disease***, mice with angiotensin II or doxorubicin induced heart failure had decreased cardiac fibrosis, hypertrophy, improved cardiac function and decreased inflammation following treatment with navitoclax (121, 122). Aged mice treated with navitoclax had a significantly reduced number of telomere associated foci, fibrosis and reduced SC, but there was no difference in cardiac ejection fraction or left ventricle mass compared with controls (123). These findings were also supported in a mouse model of ischemia-reperfusion cardiac injury, where mice treated with navitoclax had significant reduction in pro-inflammatory, profibrotic, and anti-angiogenic cytokines (interferon gamma-induced protein-10, TGF-β3, interleukin-11, interleukin-16, and fractalkine) (124). Senotherapeutic clearance of β-cells in obese metabolically dysregulated transgenic mice treated with navitoclax had decreased SASP factors, increased glucose tolerance and increased β-cell metabolism. This study suggests that pancreatic β-cell senescence may also play a role in peripheral insulin intolerance and predisposition to type 2 diabetes (125).

With relation to age related ***neurodegenerative disorders***, navitoclax treated mice had increases in neurogenesis of hippocampal neuronal precursors and increases spatial memory (126). Cellular senescence also plays a role in chronic skin diseases like scleroderma (127–130) and psoriasis (131, 132) by promoting inflammation, fibrosis, and tissue dysfunction. One study used young and old hairless mice treated with navitoclax showed selective clearance of senescent dermal fibroblasts (133). Furthermore, aged mouse skin treated with navitoclax had increased collagen density, epidermal thickness, proliferation of keratinocytes, and decreased SASP factors such as MMP-1 and IL-6 (133).

Relevant to ***pulmonary disease***, a mouse model of idiopathic pulmonary fibrosis found that radiation induced pulmonary fibrosis could be reversed by the clearance of senescent type II pneumocytes with Navitoclax treatment (134). However, secondary effects following clearance of heavy SC burden in chronic disease have been described, such as vessel remodeling, increased right ventricular systolic pressure, and increased cardiac hypertrophy index in rodents following navitoclax treatment (24). These findings indicate that consideration should be given to the disease severity and potential for systemic reaction following senotherapeutic treatment, particularly in more chronic disease processes. With respect to ***renal disease***, navitoclax has been shown to specifically target senescent proximal tubular epithelial cells and resulted in improved renal function with decreased renal fibrosis demonstrated in mice (135).

The effects of navitoclax on clinical ***bone health*** are mixed when it comes to osteoporosis and bone loss with age. One study indicated that navitoclax decreased the senescent cell burden and decreased the trabecular bone in aged mice by up 60.1% in females (136). With increased cytotoxicity, use of navitoclax in vivo should be carefully considered.

### Luteolin

Luteolin is a flavonoid found in celery, broccoli, dandelion, carrots, and olive oil (137) that exerts senotherapeutic properties through modulation of sirtuin 1 (SIRT1) and p53. Luteolin has largely been studied in mouse auditory cells (House Ear Institute-Organ of Corti 1) with 50 % efficacy in reversing senescence induced with hydrogen peroxide, although it appears less effective in reducing senescence in MEF (49, 51, 52). Through the upregulation of SIRT1, luteolin effectively protects against senescence induced by hydrogen peroxide (52). This mode of action was confirmed when the knockout of SIRT1 resulted in induced senescence. Luteolin also protected cells from peroxide induced senescence by decreasing p53 phosphorylation and p21 expression (52).

### Curcumin

Curcumin is a senotherapeutic that selectively targets apoptotic pathways such as nuclear factor NF-kB, mitogen-activated protein kinases (MAP-kinase), p53, nuclear factor erythroid 2-related factor 2 (NRF2), AKT, COX-2 and EGFR (49, 53, 54), although its effect is relatively weak relative to other senotherapeutics due to limited bioavailability (54, 138). When attempting to concentrate curcumin above 10 μM, it exhibits genotoxic and cytotoxic effects. To mitigate these toxicity risks, a ***curcumin analog*** ***EF24*** has shown promise against SC through the proteasomal degradation of Bcl-2 family proteins and production of reactive oxygen species (ROS), although further investigation is indicated (55).

### A1331852 and A1155463

The compounds A1331852 and A1155463 are both BCL-XL inhibitors, but with a lower relative risk of BCL-2 mediated neutrophil toxicity compared to navitoclax (48, 117). Treatment with A1331852 and A1155463 resulted in apoptosis, shown through enhanced caspase3/7 activity, of senescent HUVECs and IMR90 cells, but not preadipocytes (48). Further mechanistic insights of A1331852 have shown caspase-dependent apoptosis of senescent chondrocytes and increased expression of a pro-apoptotic Bcl-2 family member called BAK (139). Utilizing live cell fluorescence resonance imaging, A1331852 was shown to interfere with binding of BCL-XL (139). *In vivo*, treatment of genetically modified mice with A1331852 resulted in clearance of 80% of senescent cholangiocytes, reduced expression of fibrosis-inducing growth factors, and subsequent reduction in liver fibrosis (140).

### Heat shock protein 90 inhibitors

Heat shock protein 90 (HSP90) inhibitors influence protein stability and function, impacting p53′s ability to regulate apoptosis and DNA repair (141). ***Geldanamycin and tanespimycin*** ***(17-AAG)*** reduce SC viability, although geldanamycin in not particularly water soluble, while ***alvespimycin (17-DMAG)*** is (53). The targeted effects of geldanamycin and Tanespimycin are specific to HSP90. All HSP90 inhibitors have a dose-dependent senotherapeutic effect that is not cell type-specific (53). The *in vivo* effect of alvespimycin treatment in age related symptoms in mice, resulted in a significant reduction in kyphosis, dystonia, tremor, loss of forelimb grip strength, coat condition, ataxia, gait disorder, and overall body condition when compared to sex matched untreated mice (53). Similarly, azythromycin has been briefly studied for its ability to reduce senescent human fibroblasts by 97% (142). However, many of the senotheraputics in the HSP90 inhibitor class were initially developed and FDA approved for their antimicrobial action. Therefore, selection of these senotherapeutics must be made with antimicrobial stewardship in mind.

### Piperlongumine

Piperlongumine is a therapeutic agent often paired with chemotherapeutics because of its established pro-apoptotic properties (143). The precise mechanism of piperlongumine in unknown (56). It was previously thought that piperlongumine promoted the production of reactive oxygen species; however, it has since been proven to be an ROS-independent mechanism (56). Piperlongumine has been shown to promote caspase activity and kill senescent human WI-38 fibroblasts (56). Piperlongumine, has been assessed in an *ex vivo* goat osteoarthritis model, demonstrating decreased p53 and p16 gene and protein expression in senescent chondrocytes in a concentration-dependent manner following treatment (57). Furthermore, piperlongumine treatment rescued the oxidative stress cause by IL-1β in cartilage explants, indicating that it has potential benefit to rescue senescent chondrocytes in OA (57).

### FOXO-related peptide

The FOXO-related peptide was engineered to be a permeable peptide in p53-interaction domain in FOXO4. Treatment with this synthetic peptide induces apoptosis in senescent fibroblasts through the nuclear exclusion of p53 (58). In a concentration-dependent manner, FOXO-related peptide reduced the viability of senescent by 11.73-fold compared with control IMR90 cells (58). Importantly, the targeted design of FOXO-related peptide allows it to be safe to normal cells. *In vivo*, the FOXO-related peptide restored fitness, hair density, and renal function in aged mice (58).

### Nutlin-3a

Nutlin-3a acts as an inhibitor of MDM2, a ubiquitin ligase responsible for downregulating p53 (59). Interestingly, Nutlin-3a has been described as both a senotherapeutic (60, 144) and a senescence inducing agent (59, 145, 146). Originally studied for its anti-cancer properties, Nutlin-3a has proven effective in inducing apoptosis in carcinomas (147, 148), melanomas, T-cell lymphoma (149) and adult T-cell leukemia (145). The senescence inducing properties of Nutlin-3a have been described in normal human and mouse fibroblasts (146, 150), non-small cell lung cancers (151), adult T-cell leukemia cells (145), cutaneous T-cell lymphoma (149), glioblastomas (152), renal carcinoma (153). It is important to note that a dose dependent cytotoxic effect has been described in both senescent and non-senescent melanoma cells at concentrations ranging from 2.5 to 10 μmol/L *in vitro* (60). Further studies are needed to evaluate the pharmacodynamics and pharmacokinetics of Nutlin-3a in different cellular phenotypes. *In vivo*, The senotherapeutic nutlin-3a has been investigated as a treatment for age-related macular degeneration (144). Treatment of a mouse model with nutlin-3a showed a significant recovery of visual function (144) and ameliorated retinal degeneration (154).

### Cardiac glycosides

Another previously established drug class, cardiac glycosides has recently been described for their senotherapeutic properties (61, 62, 155). This class of drug is FDA approved for the treatment of heart failure and arrythmias such as atrial fibrillation (156, 157). Acting with Na+/K+ ATPase pump inhibition, cardiac glycosides target SCs with higher H+ concentrations and slightly depolarized membranes (62, 155). On the *in vitro* scale, the cardiac glycoside digoxin had significant senotherapeutic activity against A549 tumor cells, primary human BJ fibroblasts, and osteoarthritic chondrocytes (62). However, efficacy in mouse embryo fibroblasts was not seen (62). Treatment with Digoxin had significant senotherapeutic activity in a mouse model of IPF (62). However, research on the senotherapeutic uses of cardiac glycosides is still in its infancy and further investigation is needed to determine efficacy against different senescent phenotypes *in vivo*.

### Aspirin

The non-steroidal anti-inflammatory, aspirin, has an established repertoire in reducing endothelial senescence (158–160). Recently, aspirin has been investigated for its senotherapeutic abilities to ameliorate the long term effects on patients that have received chemotherapy and radiation (63). This study found that aspirin suppresses p53 and p21 accumulation in doxorubicin induced senescent human fibroblasts and murine embryonic fibroblasts (63). Cyclooxygenase 2 (COX2) knockout mouse embryonic fibroblasts that underwent the same treatment had a significant reduction in p53 accumulation (63). This data suggests that aspirin's senotherapeutic activity is through the inhibition of COX2. *In vivo*, aspirin treatment significantly reduce amyloid-β_42_ induced senescent neuronal cells by upregulating sirtuin-1 (SIRT1), a key regulator in cell aging (161). Aspirin has also demonstrated senotherapeutic potential in doxorubicin-treated mouse models through the reduction of SA-ß-Gal staining in liver, spleen, pancreas, and lung tissues when compared to controls (63).

### Second generation senotherapeutics

Following the identification of readily available “first generation” senotherapeutics, the so-called “second generation” of these drugs have been more recently identified and engineered compounds (Table 2). The information available pertaining to preclinical evidence for their application in veterinary medicine is summarized below.

**Table 2:** “Second generation” senotherapeutic drugs.

**2nd generation**					
**Senotherapeutic**	**Mode of action**	**Efficacy**	**Dose**	**Notes**	**References**
Galacto-oligosaccharide-coated nanoparticles with toxic cargos	Drug encapsulated beads coated w/an oligosaccharide (targeting β-galactosidase) are taken into SC lysosomes, and the drug is released via exocytosis	1. Gal-encapsulated doxorubicin = higher levels of apoptosis in senescent cells 2. Gal-encapsulated navitoclax = higher levels of apoptosis in senescent cells	100 mg of drug/gram of beads ~30 mg of drug is released per gram of beads	Decreased systemic side effects compared to systemic navitoclax administration	(162)
Vaccines/immunomodulation	Inducing or modifying immune responses to SC	1. CD153 vaccination: potentially removes senescent T-cells from high-fat diet-induced obese C57BL/6J mice 2. Oncolytic Measles Vaccine Virus can decrease SC in tumors (proof of concept) 3. sPD1-expressing senescent tumor cell vaccine induced anti-tumor response		Early trials and development	(163–168)

### Oligosaccharide coated nanoparticles

The development of oligosaccharide coated nanoparticles containing drug encapsulated beads such as doxorubicin or navitoclax are capable of inducing apoptosis in senescent cells (162). Direct targeting through endocytosis allows these drugs to be delivered intracellularly with minimal reported systemic side effects to date (162).

### Senotherapeutic vaccines

Senotherapeutic vaccinations have evolved at the intersection of oncology and immunotherapy. Chemotherapy-induced tumor senescence has been demonstrated to limit further tumor growth and can allow for immunomodulation through vaccination against the static tumor cell type, leading to the development of senotherapeutic vaccination (163–166). A murine model of senescence-related aging implemented a CD153 vaccine to effectively clear senescent T-cells (164). In another mouse model, transcriptomic analyses were used to evaluate vascular endothelial cells for senescent cell markers which identified transmembrane glycoprotein nonmetastatic melanoma protein B (GPNMB) as a sero-antigen candidate (165). Following vaccination with GPNMB, improvement in aging phenotypes and male mouse lifespans were noted (165). While these targeted approaches are promising, additional preclinical modeling and Phase 1 clinical testing are needed to ensure their safety and efficacy (164–168).

### CAR-T targeting senescence

Recent attention has focused on chimeric antigen receptor T cell (CAR T cell) modulated treatments targeting SC accumulation. The urokinase-type plasminogen activator receptor (uPAR) was identified as an expressed SC surface antigen, making it a potential target for immunomodulatory senotherapeutics (169). Treatment with uPAR-specific CAR T cell therapy demonstrated efficacy in reducing liver fibrosis and improving treatment outcomes in mouse models of lung adenocarcinoma, which warrants further investigation (169).

## Human clinical trials evaluating senotherapeutics

At present, human clinical trials are underway to assess the safety and effectiveness of senotherapeutic agents. These trials cover a diverse spectrum of medical conditions, including idiopathic pulmonary fibrosis, hematopoietic stem cell transplants, chronic diabetic kidney disease, childhood cancer survivors, age related osteoporosis, Alzheimer's disease, frailty, macular degeneration, osteoarthritis, and viral infections such as COVID-19. While initial findings indicate promising results in terms of medication safety and patient tolerance, many of these clinical trials are advancing to phase two and necessitate a randomized, blinded, placebo-controlled study design. Current trials are summarized in Table 3.

**Table 3:** Clinical trials involving senotherapeutic, as listed on clinicaltrials.gov (as of December 2023).

**Disease**	**ClinicalTrials.gov ID**	**Senotherapeutic**	**Status**
Age related frailty	NCT03430037	Fisetin	Recruiting
Age related frailty	NCT03675724	Fisetin	Recruiting
Alzheimer's	NCT04063124	D, Q, D+Q	Complete (78, 170)
Alzheimer's	NCT04685590	D + Q	Recruiting
Alzheimer's	NCT05422885	D + Q	Active
Alzheimer's	NCT04785300	D + Q	Enrolling
Arterial endothelial dysfunction	NCT06133634	Fisetin	Recruiting
Carpal tunnel syndrome	NCT05416515	Fisetin	Recruiting
Childhood cancer frailty	NCT04733534	D + Q, Fisetin	Recruiting
Chronic kidney disease	NCT02848131	D vs Q	Enrolling (77)
Coronary artery disease	NCT04907253	Q	Active
COVID-19	NCT04771611	Fisetin	Completed
COVID-19	NCT04537299	Fisetin	Enrolling
COVID-19	NCT04476953	Fisetin	Enrolling
Diabetic macular edema	NCT04857996	UBX1325	Complete
Diabetic macular edema or age-related macular degeneration	NCT04537884	UBX1325	Complete
Fatty liver disease	NCT05506488	D + Q	Recruiting
Femoroacetabular Impingement	NCT05025956	Fisetin	Recruiting
Healthy Skeletal Muscle	NCT04313634	D, Q, Fisetin	Active
Hematopoietic stem cell transplant survivor	NCT02652052	D, Q	Recruiting
Idiopathic Pulmonary Fibrosis	NCT02874989	D +Q	Complete (14, 171)
Long COVID-19	NCT04903132	-	Recruiting
Healthy vs. Obesity	NCT05653258	D, Q	Not yet Recruiting
Osteoarthritis (Knee) OA	NCT04129944	UBX0101	Complete
Osteoarthritis (knee) OA	NCT04229225	UBX0101	Complete
Osteoarthritis (knee) OA	NCT04210986	Fisetin	Complete
Osteoarthritis (knee) OA	NCT04815902	Fisetin	Active
Osteoarthritis (knee) OA	NCT03513016	UBX0101	Complete
Osteoarthritis (meniscal repair)	NCT05505747	Fisetin	Not yet Recruiting
Osteoarthritis (OA)	NCT05276895	D +/- Fisetin	Suspended
Osteoporosis	NCT06018467	D + Q	Recruiting
Post-cancer frailty	NCT06113016	Fisetin	Not yet Recruiting
Post-cancer frailty	NCT05595499	Fisetin	Recruiting
Sepsis	NCT05758246	Fisetin	Recruiting

## Senotherapeutic potential in veterinary species

**Figure 3:**
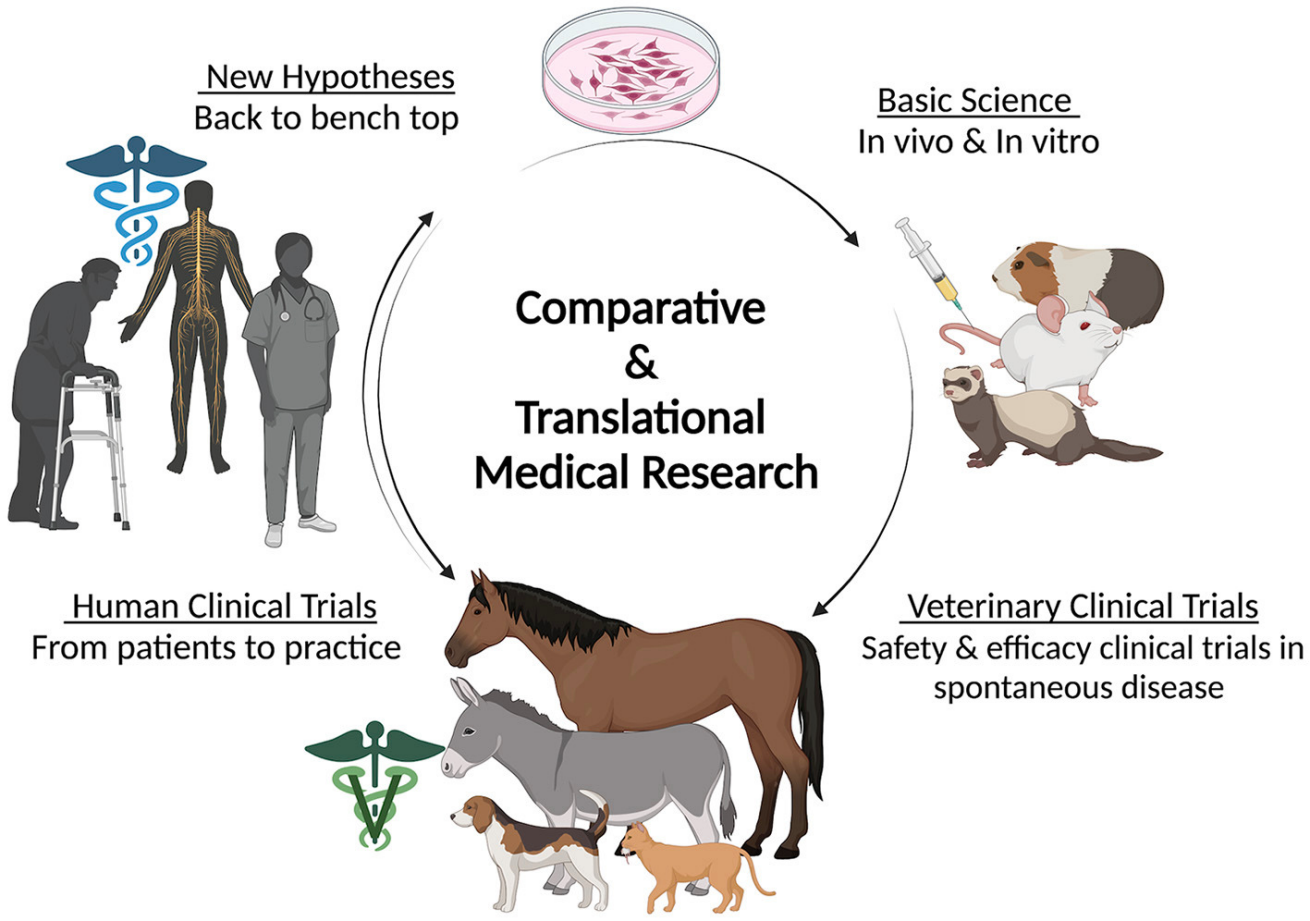
Diagram depicting the circular flow of comparative and translational medical research including the contribution of veterinary species in clinical trials. Created with BioRender.com.

**Figure 4:**
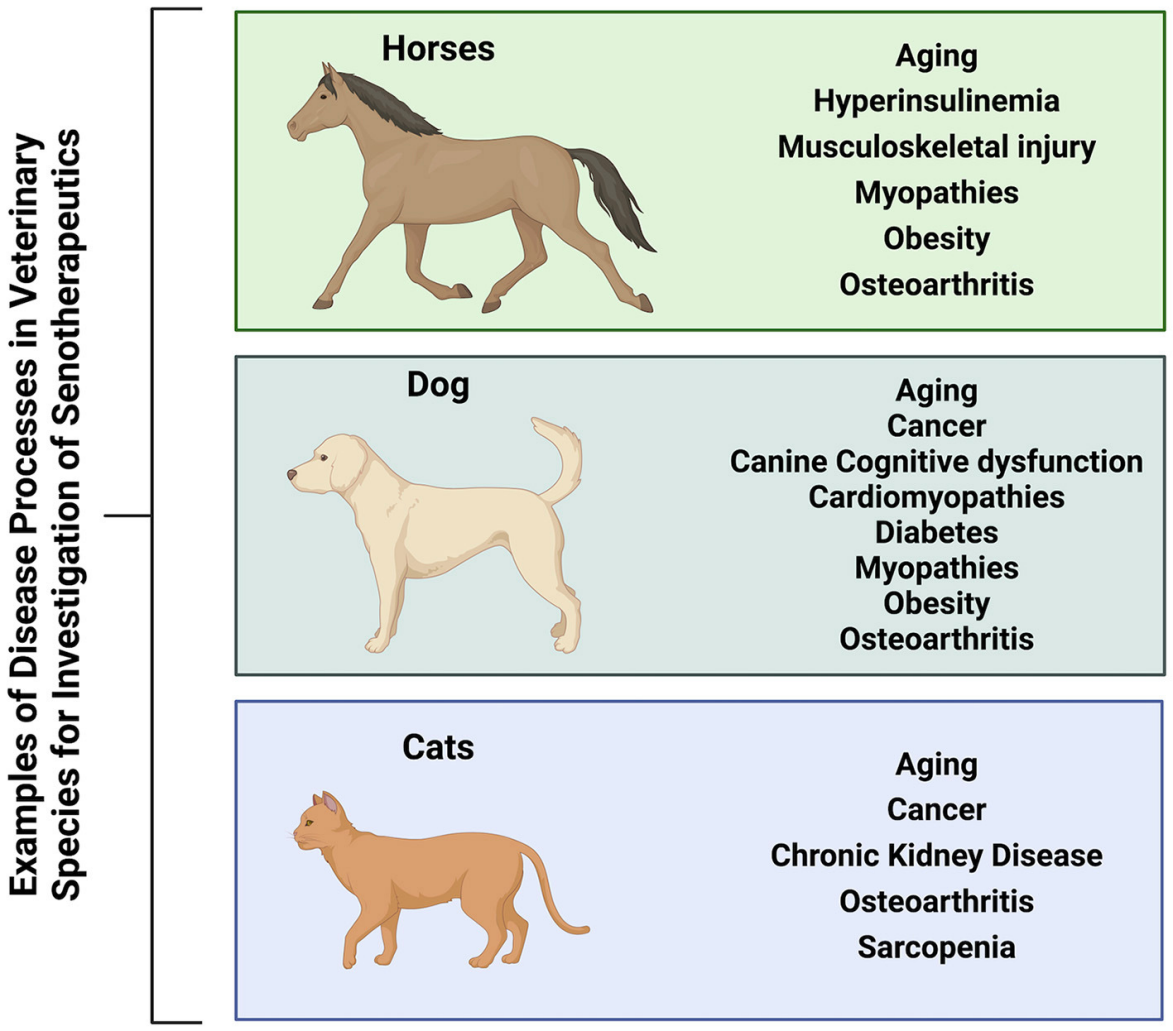
Veterinary species (horses, dogs, and cats) suffer from many similar disease processes to humans that may benefit from treatment with senotherapeutics, serving as preclinical naturally occurring disease models to also provide translational data for future human clinical trials. Created with BioRender.com.

The use of first generation senotherapeutics (e.g., dasatinib) in veterinary medicine has largely been studied in the context of neoplasia (31, 32, 172–174). Moving forward, evaluation of senotherapeutics to specifically target SC in the context of naturally occurring veterinary diseases presents a translational opportunity to explore the long-term safety and efficacy of senotherapeutics in spontaneous disease models (Figure 3). Examples of potential applications of senotherapeutics in veterinary medicine include treatment of osteoarthritis which is prevalent in dogs, horses and cats (175, 176), or more specific disease processes such as idiopathic pulmonary fibrosis in West Highland Terriers (177, 178), canine cardiomyopathies (179–181), or renal disease and sarcopenia in cats (182–185) (Figure 4). These collaborative prospects offer a new avenue to bridge the gap between *in vivo* rodent models and clinical trials in people, while simultaneously benefiting veterinary species suffering from similar disease processes.

### Osteoarthritis in veterinary species

To highlight a few disease processes for example, osteoarthritis (OA) is found to be a common cause of pain and lameness in horses (186, 187). Approximately 33% of the equine population overall in the US is estimated to be affected by OA (188), and that prevalence climbs to 50% at 15 years of age and 80 to 90% in horses over 30 years of age (189–191). Therefore, OA risk is highly associated with aging, which has been similarly reported in humans (175, 176). In conditions such as osteoarthritis, rheumatoid arthritis, and age-related frailty, senescent cells can accumulate in musculoskeletal tissues. Transplantation of SC has precipitated an OA-like phenotype in mice when compared to transplantation of non-SC (192). A recent study evaluating synovial fluid in healthy horses and horses with OA found an increased number of senescent mesenchymal stem cells (193). The senescent cells displayed impaired chondrogenic differentiation (193) when compared with a non-senescent cell population, making them potential targets for senotherapeutics. Murine models of rheumatoid arthritis (RA) found that dasatinib was protective against RA by inhibiting osteoclastogenesis through immunomodulatory effects (194). Additional murine studies have shown that SC interact with synovial cells and that dasatinib and/or quercetin have been effective in ameliorating cartilage damage and pain due to OA, as well as alleviating post-menopausal osteoporosis (73, 75, 195, 196). These findings collectively support further investigation of senotherapeutics as a potential disease modifying treatment in OA.

### Obesity in veterinary species

Senescent cells have been found to accumulate in adipose tissue which is associated with chronic low-grade inflammation and insulin resistance (17, 76, 197–200). Senotherapeutics have been shown to reduce senescent cell burden in diet-induced obesity, to alleviate metabolic dysfunction, and to restore the capacity of preadipocytes to differentiate into functional insulin-responsive fat cells (88). Obesity in companion animals is prevalent in the US (e.g., 20–45% of riding horses) and has increasingly been recognized as a factor associated with insulin resistance and OA progression. Implementation of senotherapeutics in the population of horses commonly treated for OA has potential benefit to simultaneously alleviate multiple co-morbidities (2, 201–203). Displaying an age-related phenotype, metabolic dysregulations may precipitate insulin resistance (204, 205). Hyperinsulinemia induces human hepatocyte senescence (206), which has been shown to be attenuated by dasatinib and quercetin. Interestingly, increased cellular senescence has also been observed in equine adipose-derived stem cells in horses with equine metabolic syndrome and is associated with impaired antigen stability and clonogenic potential (207). SC have altered metabolism compared to non-SC (208) and likely contribute to impaired fat metabolism and insulin resistance. Elimination of these cells may aid in healthy weight loss and return to metabolic homeostasis, potentially improving treatment of insulin resistance and associated disease processes (76).

### Cardiac disease in veterinary species

Senescent cells have been found to contribute to the progression of atherosclerosis, arterial stiffness, and heart failure in human cardiovascular disease (120, 122, 209–211). The risk of heart disease increases with age and precipitates hemodynamic instability. Senotherapeutics have been investigated in canine myxomatous mitral valve disease (212). Valve interstitial cells that were treated with quercetin or quercetin plus dasatinib showed a decrease in SC and p53 expression (212). The decrease in SC is achieved through PI3K/AKT/mTOR antagonism which facilitates the reversal of myofibroblast senescence and promoting autophagy (213). Therefore, current therapeutic approaches for mitral valve disease could be expanded to include senotherapeutics which may slow disease progression (212).

### Renal disease in veterinary species

Senescent cells can accumulate in renal tissue, contributing to inflammation, fibrosis, and impaired function (100, 135, 214). Eliminating dysfunctional cells may reduce inflammation, decrease fibrosis, slow down the progression of kidney damage, and preserve renal function (100, 214). Spontaneous chronic kidney disease (CKD) is well-described in cats (182–185). Feline patients suffering from CKD have renal senescence, telomere shortening, and nitrosative stress in renal cells (215). With similar pathologic findings to humans (216, 217), they represent a naturally occurring model for study of senotherapeutics (215). *In vitro* studies utilizing feline renal cells have shown a dose dependent correlation with radiation and SC (218). Further studies can utilize this data to understand the etiopathology of radiotoxicity induced renal senescence and investigation of senotherapeutics *in vivo* in this context (Figure 4).

### Limitations of senotherapy in veterinary species

Despite the potential benefits, there are prominent limitations that call for collaborative research to fully understand the breadth and depth of senotherapy in veterinary species. Studies in laboratory species and human clinical trials are still examining the full extent of side effects and long-term effects of senotherapeutics. It remains crucial to also examine the impact this drug class has on healthy cells and to not lose focus on the unknown effects on cell and tissue homeostasis. This raises concerns for off-target effects and toxicity in veterinary species since there are metabolic nuances depending on which species is studied. SC have different markers and characteristics depending on the tissue and the cause of senescence. The heterogeneity in SC makes it difficult to develop universally effective protocol, especially when considering effective dosing across species. Lastly, it is important to recognize that senescent cells could become resistant to these therapies. Similar with anthelmintic use and antibiotic stewardship, judicious use of medication relies on accurate diagnostics, clinical monitoring, and continued research.

## Discussion

Senotherapeutics attenuate tissue inflammation and restore progenitor cell function to delay, prevent or alleviate symptoms in multiple age-related diseases (3). The first generation senotherapeutics have been largely deemed safe and with varying degrees of efficacy (1, 135, 219, 220), with the exception of navitoclax and curcumin that have demonstrated cytotoxicity (28, 31, 39, 40). Optimal dosage and duration for various disease processes have not been fully explored. Although short intermittent dosing regimens appear effective and offer clinical translation with fewer negative off-target effects compared to sustained administration with currently available drugs. Administration for extensive periods may consequently deplete cell types necessary for remodeling (1, 3, 49, 221, 222). The majority of senotherapeutic pre-clinical trials have been conducted in induced murine models (49, 53, 83, 101, 114, 135). Further development of senotherapeutics for use in naturally occurring companion animal models of disease may inform future human clinical trials regarding pharmacokinetic, pharmacodynamic and interactions with other drugs that may be administered concurrently (223–226).

Treatments targeting senescent cells and the SASP have broad potential implications in the field of veterinary medicine as the hallmarks of aging (6) are highly conserved across species (227, 228). These hallmarks include DNA damage (229), telomere shortening (230, 231), aberrant proteostasis (232), epigenetic modifications (233), altered nutritional signaling (234, 235), cell senescence (193, 213, 215, 231, 236), stem cell depletion (237), mitochondrial dysfunction (238, 239), and abnormal inflammatory signaling (240, 241). Treatment of age-related diseases in veterinary species may further serve to bolster preclinical evidence for use in humans. To date, the majority of preclinical or *in vitro* studies have largely focused on investigating dasatinib, quercetin, or fisetin (1, 171). Dasatinib is well known in the oncology realm and has been used to treat a variety of human, canine, and feline cancers (64, 65, 172–174, 242). Additionally, quercetin has also been beneficial in treating canine neoplasia including osteosarcoma cells (243, 244). Similar to humans, sarcopenia (185, 245–247) and decreased bone density (248, 249) is commonly associated with age in both felines and canines. The use of senotherapeutics for Alzheimer's disease is being evaluated (74, 78, 170, 250) and canine or feline cognitive dysfunction may serve as a comparative naturally occurring model (249, 251–253). Osteoarthritis is also significantly linked with age and cellular senescence (193, 196). Due to similarities in cartilage thickness and joint volume, equine models are well suited comparisons of human osteoarthritis (175, 176, 254). Greater recognition of similarities in animal preclinical models to human aging related disorders has the potential to advance the field of senotherapeutics to the benefit of both humans and veterinary species.

The current state-of-play in the field of senotherapeutics presents multiple avenues for future research. Further development of species- and tissue-specific biomarkers for senescent cell abundance, SASP mediators, and senescent phenotypes (i.e., senotype), a field termed “gerodiagnostics” will be critical to fully understand the effects of senotherapeutics and reduce off-target effects with their administration (1, 3, 15). Generation of comprehensive atlases of senescent cells that arise during aging and disease states across multiple tissue types and specific to the target species of interest are warranted (221). Toward this goal, the National Institute of Aging (NIA) has established a common fund's cellular senescence network (SenNet) program to generate atlases for humans and mice (221), to facilitate identification of senotype specific biomarkers that will help to identify the therapeutic window for senotherapeutic interventions and to guide dosage, timing, and duration of senotherapeuthic treatments in the aging population. Future directions for new drug development may include evaluation of clearance of senescent cells using genetic and epigenetic approaches or interventions that modulate SASP (1, 3, 15). Correlation of interventions with more specifically defined veterinary gerodiagnostics and development of therapeutic strategies targeting fundamental aging processes such as dietary changes and exercise will further the field. Expanded clinical trials to ensure safety, benefit, and target engagement first in serious disease processes followed by other senescence associated disorders are indicated. Results of ongoing clinical trials will yield insights and informative data into the role of cellular senescence as a therapeutic target for age-related disorders (1, 3, 15). Evaluation of senotherapeutics in different age groups will identify limits of biomarkers and therapeutic benefit in different species and signalments. Future clinical trials could facilitate identification of systemic markers that could be associated with senotherapeutic responsive individuals given inter-individual variability in aging (e.g., circulating SASP factors, cytokines such as TGF-ß). Finally, more comprehensive investigation of the mechanisms of action of senotherapeutics is indicated as proposed mechanisms have other functions outside of addressing senescence, thus confounding the contributions of each response during tissue repair process and aging. Addressing these scientific and regulatory challenges will be critical if senotherapeutics are to be used outside of clinical trials and in veterinary medicine.

## Conclusions

Cellular senescence is considered a “double-edged” sword in the balance of disease and health states (4), and addressing states of immunosenescence, both systemically and locally, represents a novel treatment of age-related diseases in veterinary medicine (255). Senotherapeutic drugs identified via bioinformatic analyses present a novel therapeutic strategy to selectively clear senescent cells with broad implications to aging related disorders. Dasatinib, quercetin and fisetin represent the most studied compounds to date and are currently under investigation in human clinical trials. Further exploration of senotherapeutic applications in companion animals, including enhanced understanding of mechanism of action and investigation of route of delivery, bioabsorption, and potential off-target effects represents a new frontier to extend healthspan in veterinary patients.

## Author contributions

ZW: Data curation, Investigation, Methodology, Writing—original draft, Writing—review & editing. LC: Conceptualization, Data curation, Methodology, Writing—original draft, Writing—review & editing. SD: Conceptualization, Data curation, Formal analysis, Funding acquisition, Investigation, Project administration, Resources, Software, Supervision, Visualization, Writing—original draft, Writing—review & editing. LP: Conceptualization, Data curation, Formal analysis, Funding acquisition, Investigation, Methodology, Project administration, Resources, Software, Supervision, Validation, Visualization, Writing—original draft, Writing—review & editing.
